# DRCM: a disentangled representation network based on coordinate and multimodal attention for medical image fusion

**DOI:** 10.3389/fphys.2023.1241370

**Published:** 2023-11-03

**Authors:** Wanwan Huang, Han Zhang, Yu Cheng, Xiongwen Quan

**Affiliations:** College of Artificial Intelligence, Nankai University, Tianjin, China

**Keywords:** medical image, image fusion, coordinate attention, multimodal attention, exclusive feature, deep learning

## Abstract

Recent studies on medical image fusion based on deep learning have made remarkable progress, but the common and exclusive features of different modalities, especially their subsequent feature enhancement, are ignored. Since medical images of different modalities have unique information, special learning of exclusive features should be designed to express the unique information of different modalities so as to obtain a medical fusion image with more information and details. Therefore, we propose an attention mechanism-based disentangled representation network for medical image fusion, which designs coordinate attention and multimodal attention to extract and strengthen common and exclusive features. First, the common and exclusive features of each modality were obtained by the cross mutual information and adversarial objective methods, respectively. Then, coordinate attention is focused on the enhancement of the common and exclusive features of different modalities, and the exclusive features are weighted by multimodal attention. Finally, these two kinds of features are fused. The effectiveness of the three innovation modules is verified by ablation experiments. Furthermore, eight comparison methods are selected for qualitative analysis, and four metrics are used for quantitative comparison. The values of the four metrics demonstrate the effect of the DRCM. Furthermore, the DRCM achieved better results on SCD, Nabf, and MS-SSIM metrics, which indicates that the DRCM achieved the goal of further improving the visual quality of the fused image with more information from source images and less noise. Through the comprehensive comparison and analysis of the experimental results, it was found that the DRCM outperforms the comparison method.

## 1 Introduction

Medical imaging is widely used in medical diagnosis. It can show pathological tissues that cannot be observed by the naked eye, so it can assist doctors in making accurate judgments on the condition and reduce the possibility of misdiagnosis (Iglehart, 2006). Medical imaging mainly includes ultrasound, computed tomography (CT), fluorescence examination, and positron emission tomography (PET) (James and Dasarathy, 2014). CT can detect abnormalities in the brain, neck, chest, abdomen, and pelvis. However, in some areas, there are too many artifacts of the bones, affecting the display effect of soft tissue lesions (Burns et al., 2017; Dong et al., 2019). MRI can be used for multi-plane imaging, which makes up for the shortcoming of CT of being unable to be used for direct multi-plane imaging. However, it has a poor effect in showing lesion calcification and bone cortex, so it is not suitable for observing fractures. However, characteristic intracranial calcification is often helpful for qualitative diagnosis. PET has the advantages of high sensitivity, three-dimensional imaging, being non-destructive, and accurate positioning, which is important in early tumor diagnosis. The specificity of PET is very high, and the qualitative diagnosis can be made according to the characteristics of the hypermetabolism of malignant tumors (Wang et al., 2018a).

Medical image fusion is the technique to handle the aforementioned disadvantage of medical imaging, which synthesizes multiple images into a new image (Du et al., 2016). Medical image fusion generally includes the integration of anatomical images with other anatomical images as well as the integration of anatomical images and functional images. The most common anatomical image pair is CT–MRI, while the anatomical and functional image pairs include MRI–PET and MRI–SPECT. The fusion of anatomical images consolidates diverse anatomical information from different modalities into a single image, thereby facilitating medical information analysis for healthcare professionals. The fusion of anatomical and functional images can integrate anatomical and functional information into one image, enabling doctors to assess metabolic data alongside precise visualization of tissues and organs. The fusion image can make use of the correlation and information complementarity of the original image, to have a more comprehensive and clear description of the scene so as to be more conducive to the diagnosis of the disease. In this paper, we investigate how to accurately disentangle features and further address common redundant information problems in multimodal medical image fusion. The proposed method significantly enhances the visual quality of the fused image with clearer edges and richer detail information, surpassing the performance of state-of-the-art methods.

So far, extensive research efforts have been dedicated to enhancing the quality of fused images and optimizing fusion algorithms (James and Dasarathy, 2014; Du et al., 2016). Traditional image fusion has achieved remarkable results, and as computation power has advanced, deep learning-based image fusion methods have been thoroughly investigated (Huang et al., 2020; Li et al., 2021; Azam et al., 2022). However, much of the aforementioned research focuses on improving the type, complexity, and depth of neural networks for the extraction of more profound features. Regrettably, it often neglects the inherent characteristics of the features themselves present in images from diverse modalities. The image feature reflects the detail information of the image, making the extraction of these features a pivotal component in image fusion. Although several disentangled representation methods have been introduced to extract both common and exclusive features (Li et al., 2021) from multimodal images, the disentanglement of multiple modalities in medical image fusion remains a current challenge. Furthermore, many disentangled representation methods typically rely on combination or weighted combination to fuse multimodal features, often resulting in the issue of common redundant information (Li et al., 2020), which may lead to the loss of details or blurring in the fused image.

**FIGURE 1:**
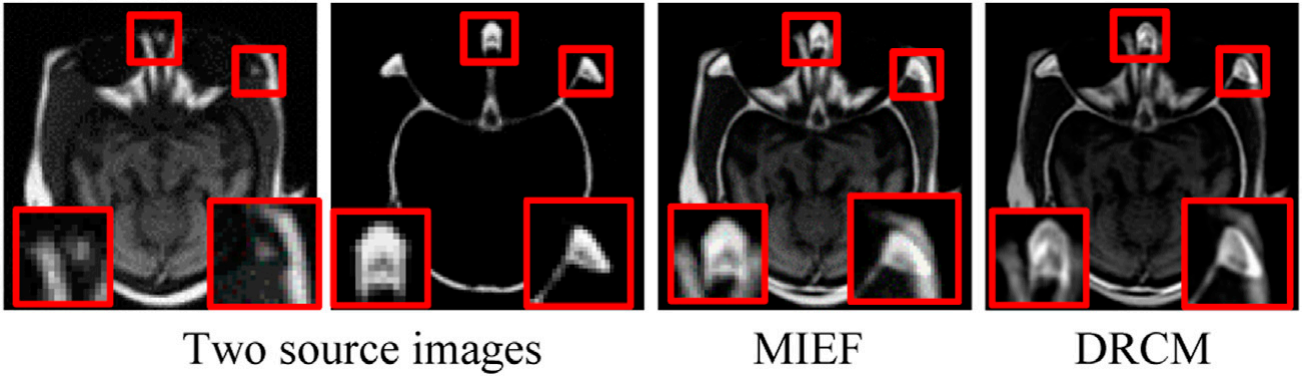
Schematic illustration of medical image fusion. From left to right: two source images (reproduced with permission from http://www.med.harvard.edu/AANLIB/home.html), MIEF (Reproduced from Huang et al. (2022a), licensed under CC BY 4.0) and the proposed DRCM. Obviously, the DRCM not only retains more information of multimodal images but also has sharper edges.

To address the aforementioned problems, we propose a disentangled representation network with coordinate attention and multimodal attention for medical image fusion (DRCM). Specifically, we design a disentangled representation network to extract the common and exclusive features from multimodal medical images, which can reflect the special characteristics of multimodal medical images. In addition, we also use the multimodal attention mechanism (Li et al., 2020) to solve the problem of common redundant information and coordinated attention (Hou et al., 2021) to further enhance the feature representation of fused images. As Figure 1 shows, two source images have different anatomical information at the same location, but MIEF (Huang et al., 2022a) encounters the problem of common redundant information, which leads to blurring of edges and contours and loss of details in its fused image, while the fused images generated by the DRCM have clearer edges and contours and can contain more information of different modalities.

Specifically, beyond previous works (Huang et al., 2022a), we propose a DRCM. More precisely, the coordinate attention is focused on the enhancement of the common and exclusive features of different modalities, and the multimodal attention is used to calculate the weights of multiple modalities by using the exclusive features. The contributions of the article are as follows:

1) A disentangled representation network based on cross mutual information and adversarial objective is adopted for extracting common and exclusive features of different modality medical images.
2) Coordinate attention captures the direction-aware and location-sensitive information of common features and exclusive features and can capture cross-channel information, which helps the network to accurately express the medical image features.
3) The multimodal attention network calculates the dynamic weight of features according to the exclusive features of each modality, avoids the interference of redundant information on the expression of exclusive features, and emphasizes the importance of exclusive features.

Overall, in order to address correct feature disentangling problems and the common redundant information problem, we propose a disentangled representation network based on coordinate and multimodal attention. The DRCM uses the cross mutual information and adversarial objective methods to extract the common and exclusive features from multimodal medical images. These features capture the special characteristics inherent in multimodal images. Furthermore, the DRCM employs the multimodal attention mechanism to address the problem of common redundant information and uses coordinated attention to further enhance the feature representation.

The remainder of this paper is structured as follows: the second part shows the related works, the third part introduces the proposed method, the fourth part verifies the method through experiments, the fifth part is the ablation experiment of the method, and the last part is the conclusion and recommendations.

## 2 Related works

### 2.1 Medical image fusion methods

Traditional methods usually fuse images in image pixels, image blocks, or image regions. The commonly used methods are bilateral filter (BF) (Shreyamsha Kumar, 2015) and guided filtering (GFF) (Li et al., 2013). Typical transform domain traditional fusion methods include the wavelet transform (WT) (Cheng et al., 2008), curvelet transform (CVT) (Ali et al., 2010), non-subsampled contourlet transform (NSCT) (Arthur et al., 2006), and Laplacian pyramid (LP) (Sahu et al., 2015). Jain and Salau (2021) and Salau et al. (2021) made a comprehensive summary of transformation, technologies, and rules of image fusion and proposed a method of image fusion using discrete cosine transform, which achieved better results. Panigrahy et al. (2020) proposed a novel weighted parameter adaptive dual-channel PCNN for PET-MRI fusion and obtained greater outperformance. Seal et al. (2018) employed the random forest and à-trous wavelet transform methods for PET–CT fusion, which performed superior in terms of visual and quantitative qualities. Some image feature representation methods also achieved an excellent fusion effect, such as the sparse representation (SR)-based image fusion method. Li et al. (2012) proposed a new dictionary learning method to realize the denoising and fusion tasks of 3D medical images. Liu et al. (2019) proposed a morphological component analysis (MCA)-based medical image fusion model. Adame et al. (2020) proposed a method with an interval-valued intuitionistic fuzzy set for CT–MRI image fusion, which is more conducive to practical clinical application. Panigrahy et al. (2023) proposed the parameter adaptive unit-linking PCNN and distance-weighted regional energy-based measure for medical image fusion, which create more informative images to specialists for disease diagnosis. However, the artificially designed fusion strategies in traditional methods are often difficult to optimize, which may result in suboptimal results and low contrast.

Since the deep learning method performs better in extracting image features, many scholars use residuals, pyramid, attention, generative adversarial network (GAN), and other methods to achieve higher-quality medical image fusion. Lahoud and Süsstrunk (2019) proposed a real-time image fusion method suitable for any number of input sources. In this method, a preprocessing network is used to generate a fusion image containing the features of multimodal images. A method based on the trained Siamese convolutional network and contrast pyramid was proposed by Wang et al. (2020a) to achieve high-quality medical image fusion. To improve the performance of an approach based on a single kind of network framework, Fu et al. (2021) attempted a multi-scale residual pyramid attention network to achieve end-to-end fusion. The aforementioned work is based on the convolution neural network. To further improve the stability and efficiency of network training, Wang et al. (2020b) used a GAN to effectively suppress artifacts and distortions in fused images. Ma et al. (2020) used the dual-discriminator conditional generative adversarial network (DDcGAN) for the fusion of infrared and visible images with different resolutions. Cheng et al. proposed a network architecture integrating an image generation module and discriminator module to generate information-rich fusion images. The image generation module is built based on dense blocks and encoder–decoder (Zhao et al., 2021). However, in the feature extraction, these deep learning-based methods often overlook the distinctions among different modalities, thus neglecting the unique characteristics inherent to each modality.

Unlike the aforementioned existing methods, our approach involves the design of a disentangled representation network aimed at extracting common and exclusive features from multimodal medical images by contrasting the information derived from various modalities. In addition, to address the common redundancy issues that may arise during the fusion process, we employed the multimodal attention mechanism to dynamically weight exclusive features. Additionally, we employed the coordinate attention to further enhance the representation of multimodal features, ultimately enhancing the visual quality of the fused image.

### 2.2 Disentangled representation

Disentangled representation is a theory aimed at modeling the underlying factors of data variations. In recent years, it has been widely used in computer vision tasks, particularly for learning the disentangled representation of input features. For instance, Sanchez et al. (2020) used a mutual information estimation method to achieve the disentangled representation of input images and completed several tasks such as image classification and segmentation. Salau and Jain (2019) divided image features into common and exclusive features of different fields and extracted the image features specifically through detailed feature classification. Wang et al. (2021) adopted the weighted parallel method to extract the common and exclusive features. Wang et al. (2022), Zhao et al. (2020), and Niu et al. (2020) extracted common and exclusive features under different modalities by maximizing the mixing of source image information. In this paper, as the goal of image fusion is to extract and fuse complementary information from multimodal images, we aim to extract the common and exclusive features between multimodal medical images. To achieve this, we designed a disentangled representation network utilizing the cross mutual information method (Huang et al., 2022a) and adversarial objective method (Sanchez et al., 2020).

### 2.3 Attention mechanisms

Attention mechanisms focus on the most important information of the input images. In the realm of computer vision, the spatial attention (Wang et al., 2018b) is dedicated to choosing features within spatial domains, while the channel attention (Guo et al., 2022) recalibrates the channel-wise features and serves as an operation of selecting the target object (Chen et al., 2017). These two attention mechanisms have their own advantages. The coordinate attention can combine the advantages of spatial attention and channel attention, which is beneficial for many visual tasks. Yi et al. (2022) combined the channel and coordinate attention to realize feature fusion for a semantic segmentation task. Zhang et al. (2021) used the coordinate attention and complex-valued method to distinguish space targets. Yang et al. (2022) employed the coordinate attention to achieve image classification. Multimodal attention (Li et al., 2020) is proposed to solve the problem of common redundant information that often occurs in multiple modalities, and this method assigns weights to multiple modalities by extracting the exclusive features. Multimodal attention has also been applied to many multimodal tasks. Liu et al. (2023) calculated the weights of different modalities for multimodal emotion recognition. Li et al. (2020) employed the multimodal attention method to assign weights to multiple modalities for click-through rate prediction.

In this article, we utilized multimodal attention to assign weights to multimodal medical images. This approach effectively solves the problem of common redundant information between modalities by utilizing the exclusive features of multiple modalities. Furthermore, we also employed the coordinate attention to enhance the features of multiple modalities, thus further elevating the visual quality of the fused image.

## 3 Proposed method

First, the procedure and framework of the DRCM method are introduced. Then, the coordinate attention performs weighted enhancement on common and exclusive features. Finally, multimodal attention is used for weighted enhancement of exclusive features.

### 3.1 Overview

**FIGURE 2:**
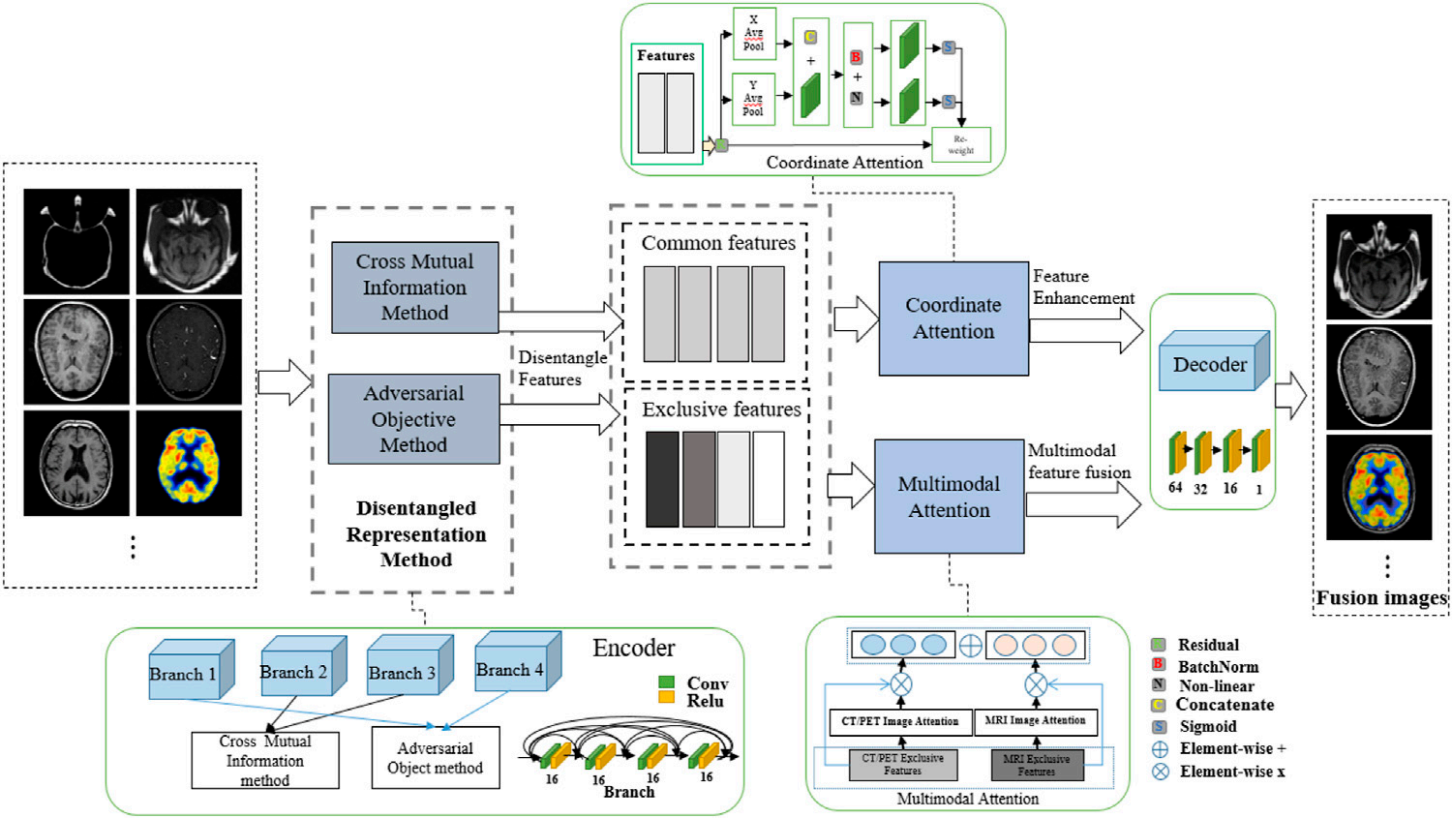
Entire network structure of the DRCM.

In this paper, a disentangled representation network with coordinate and multimodal attention is proposed. The entire procedure of the DRCM is shown in Figure 2. This model mainly includes three parts. First, we employ the mutual information estimation method (Sanchez et al., 2020) to disentangle features from image pairs, which contains the cross mutual information and adversarial objective methods and has been proven to be effective in image fusion (Huang et al., 2022a). Thus, we obtain the common and exclusive features from the multimodal images. Second, to further enhance the feature representation and improve the fusion performance, we utilize the coordinate attention to strengthen the disentangle features. Third, to exclude the influence of redundant information on multimodal data, we employ multimodal attention to dynamically weight multimodal medical images. The experiments exhibited the superiority of the DRCM compared with the state-of-the-art methods in terms of visual effects and quantitative measurement.

The designed framework of the DRCM is also shown in Figure 2. As we can see, a four-branch encoder is used to extract features, and the branches with the same structure of four convolution layers are employed to obtain common and exclusive features. Then, coordinate attention and multimodal attention are adopted to process the extracted features. Finally, according to the fusion strategy, the feature fusion is performed to reconstruct the image. According to Huang et al. (2022a), the mutual information estimation method is completed by two steps; the first step employs the cross mutual information method to extract the extract the common features $C_{1}$ and $C_{2}$ , and the second step uses the adversarial objective method to obtain the exclusive features $E_{1}$ and $E_{2}$ .

After feature extraction, coordinate attention is used to weight common and exclusive features to improve the expression of features in the network, and multimodal attention is used to dynamically weight multimodal information. By combining these weighted features and passing them through a decoder, we obtain the final fused image $I_{f}$ .

### 3.2 Feature disentangling based on mutual information estimation

The cross mutual information (Huang et al., 2022a) is used to capture common features between two different modalities, involving the mutual information between source image $I_{2}$ and $C_{1}$ , as well as the mutual information between source image $I_{1}$ and $C_{2}$ . $C_{1}$ and $C_{2}$ are the shallow features of $I_{1}$ and $I_{2}$ , respectively. It is worth noting that since we employ a 3 × 3 convolution kernel and set the stride and padding to 1, the resolution of the features is the same as that of input images. Therefore, the estimation of mutual information maximization of $I_{2}$ and $C_{1}$ and $I_{1}$ and $C_{2}$ can be computed, and the Jensen–Shannon divergence (JSD) is generally used as an approximation (Sanchez et al., 2020).

$${\hat{I}}_{\theta_{S}}^{\left(JSD\right)}\left(I_{1},C_{2}\right)=E_{p\left(I_{1}^{\prime },C_{2}\right)}\left[-\log\left(1+e^{-T_{\theta_{S}}\left(I_{1}^{\prime },C_{2}\right)}\right)\right]-E_{p\left(I_{1}^{\prime }\right)p\left(C_{2}\right)}\left[-\log\left(1+e^{-T_{\theta_{S}}\left(I_{1}^{\prime },C_{2}\right)}\right)\right],$$

$${\hat{I}}_{\theta_{C}}^{\left(JSD\right)}\left(I_{2},C_{1}\right)=E_{p\left(I_{2},C_{1}\right)}\left[-\log\left(1+e^{-T_{\theta_{S}}\left(I_{2},C_{1}\right)}\right)\right]-E_{p\left(I_{2}\right)p\left(C_{1}\right)}\left[-\log\left(1+e^{-T_{\theta_{S}}\left(I_{2},C_{1}\right)}\right)\right],$$ (1)

where $T_{\theta_{S}}$ is the statistics network with the parameter $\theta_{S}$ , which is implemented by four convolutional layers (Huang et al., 2022a), while x' denotes the feature maps of image x. Using these variables, the loss function that estimates the mutual information for common features can be obtained as follows:

$$L_{MI}^{ch}={\hat{I}}_{\theta_{S}}^{\left(JSD\right)}\left(I_{1},C_{2}\right)+{\hat{I}}_{\theta_{S}}^{\left(JSD\right)}\left(I_{2},C_{1}\right).$$ (2)

Additionally, as the features $C_{x}$ and $C_{y}$ are common between both modalities, it is important to minimize the difference between them. This can be achieved by using the $L_{1}$ distance as a constraint:

$$L_{1}=E_{p\left(C_{x},C_{y}\right)}\left\|C_{x}-C_{y}\right\|.$$ (3)

To extract the exclusive features, an adversarial objective is employed, which engages in a context between a generator and a discriminator to achieve the least mutual information between the common and exclusive features.

$$\begin{array}{l} L_{\text{adv}}=L_{adv}^{I_{1}}+L_{adv}^{I_{2}},\\ =\left[E_{p\left(C_{I_{1}}\right)p\left(e_{I_{1}}\right)}\left[\log D_{\rho }\left(C_{I_{1}},E_{I_{1}}\right)\right]+E_{p\left(C_{I_{1}},e_{I_{1}}\right)}\left[\log\left(1-D_{\rho }\left(C_{I_{1}},E_{I_{1}}\right)\right)\right]\right]\\ +\left[E_{p\left(C_{I_{2}}\right)p\left(e_{I_{2}}\right)}\left[\log D_{\rho }\left(C_{I_{2}},E_{I_{2}}\right)\right]+E_{p\left(C_{I_{2}},e_{I_{2}}\right)}\left[\log\left(1-D_{\rho }\left(C_{I_{2}},E_{I_{2}}\right)\right)\right]\right], \end{array}$$ (4)

where the discriminator with parameter $\rho_{x}$ is denoted as $D_{\rho }$ , which includes three convolution layers (Huang et al., 2022a). The adversarial loss of image *I* is represented as $L_{adv}^{I}$ .

Furthermore, in addition to the aforementioned loss functions, to obtain features for image fusion, we employ MSE and SSIM loss to achieve pixel and structural similarity between fused image $I_{f}$ and source images $I_{1}$ and $I_{2}$ .

$$L_{MSE}=\frac{1}{H\times W}\left({\left\|I_{1}-I_{f}\right\|}_{2}^{2}+{\left\|I_{2}-I_{f}\right\|}_{2}^{2}\right),$$ (5)

$$L_{ssim}=\left[1-SSIM\left(I_{1},I_{f}\right)\right]+\left[1-SSIM\left(I_{2},I_{f}\right)\right],$$ (6)

$$L_{t}=L_{MSE}+L_{ssim},$$ (7)

where H and W denote the height and width of images, respectively. $SSIM\;\left(∙\right)$ indicates the SSIM similarity of source and fusion images.

### 3.3 Coordinate attention

**FIGURE 3:**
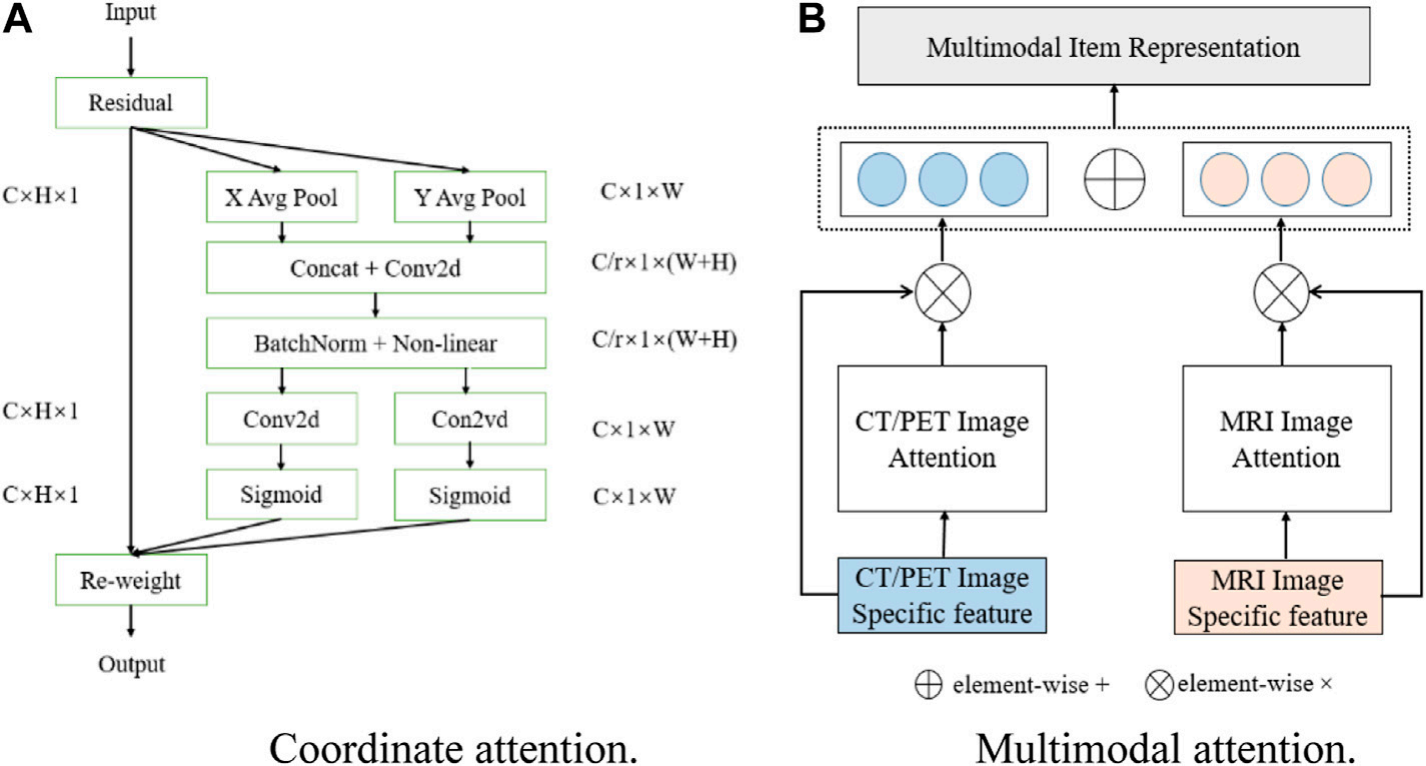
Schematic of **(A)** coordinate attention and **(B)** multimodal attention.

In computer vision, attention mechanisms mainly include spatial attention and channel attention (Guo et al., 2022), and coordinate attention can combine the advantages of channel attention and spatial attention. Different from squeeze and excitation (SE) attention (Hu et al., 2018) which directly uses global average pooling, coordinate attention factorizes the global pooling into two spatial extents of pooling kernels (H, 1) and (1, W), one of them being along the horizontal coordinate and the other being along the vertical coordinate, which is good for extracting position information in space. The schematic diagram of coordinate attention is shown in Figure 3A. The output of the *c*th channel at height h and width w is described as

$$z_{c}^{h}\left(h\right)=\frac{1}{W}\sum_{0\leq i\leq W}x_{c}\left(h,i\right),$$ (8)

$$z_{c}^{w}\left(w\right)=\frac{1}{H}\sum_{0\leq j\leq H}x_{c}\left(j,w\right).$$ (9)

Then, concatenating Eqs 1, 2 and performing the convolution operation, the expression can be expressed as

$$f=\delta \left(F_{1}\left(\left[z^{h},z^{w}\right]\right)\right),$$ (10)

where $F_{1}$ and δ denote the convolutional transformation function and non-linear activation function, respectively, and $\left[\cdot ,\cdot \right]$ denotes the concatenation operation. $f\in R^{C/r\times \left(H+W\right)}$ is the feature map, where r is the reduction ratio and set to 32 manually. Splitting f again along horizontal and vertical coordinates yields $f^{h}\in R^{C/r\times H}$ and $f^{w}\in R^{C/r\times W}$ . Then, two 1×1 convolutions $F_{h}$ and $F_{w}$ are used to transform $f^{h}$ and $f^{w}$ to obtain $g^{h}$ and $g^{w}$ , respectively, as follows:

$$g^{h}=\sigma \left(F_{h}\left(f^{h}\right)\right),$$ (11)

$$g^{w}=\sigma \left(F_{w}\left(f^{w}\right)\right),$$ (12)

where σ denotes the sigmoid activation function. Finally, $g^{h}$ and $g^{w}$ are the final weights obtained, and the resulting output can be described as

$$y_{c}\left(i,j\right)=x_{c}\left(i,j\right)\times g_{c}^{h}\left(i\right)\times g_{c}^{w}\left(j\right).$$ (13)

### 3.4 Multimodal attention

Since each modality has different effective information and exclusive features, only using common features cannot represent the exclusive characteristics of a different modality m . Therefore, multimodal attention is used to calculate the dynamic weight of exclusive features for each modality. The dynamic weighting of the exclusive feature $e_{i}$ can be written as

$$e_{i}=\sum_{m=1}^{2}{\text{atten}}_{i}^{m}⊙e_{i}^{m},$$ (14)

$${\text{atten}}_{i}^{m}=\tanh\left(W_{m}^{T}\cdot e_{i}^{m}+b_{m}\right),$$ (15)

where ${\text{atten}}_{i}^{m}$ is used to adjust the weights of different modalities and ⊙ denotes element-wise multiplication. $W_{m}^{T}$ and $b_{m}$ are the 256×256 parameter matrix and 256× 1 vector, respectively. Figure 3B shows the schematic of the multimodal attention fusion.

## 4 Results

### 4.1 Datasets and training details

In the experiments, publicly available medical images from the website of Harvard Medical School are used to train and test the network (http://www.med.harvard.edu/AANLIB/home.html). CT–MRI and PET–MRI are two commonly used modality pairs in the field of medical images (Liu et al., 2017a; Xu and Ma, 2021), and SPECT–MRI image pairs are also employed to verify the generalization of the proposed model. We select 500 CT–MRI image pairs and cropped them into over 13,000 patch pairs for training, and 500 PET–MRI image pairs are also cropped into over 13,000 patch pairs. All images have a uniform size of 256*256. In the test set, the PET and SPECT images are converted into YCbCr space, and the Y (brightness) channel is used for training. The network is trained with the five epochs in batches of four, the learning rate is 0.0001, and the optimizer is Adam. Experiments are performed on a NVIDIA GeForce RTX 2070 GPU and Intel Core i7-9700k CPU.

### 4.2 Fusion results

Eight methods are compared with the DRCM in this paper, i.e., CNN (Liu et al., 2017b), RPCNN (Das and Kundu, 2013), U2Fusion (Xu et al., 2020), DDcGAN (Ma et al., 2020), GFF (Li et al., 2013), IFCNN (Zhang et al., 2020), EMFusion (Xu and Ma, 2021), and MIEF (Huang et al., 2022a). Among these, GFF is only used in the experiments of CT–MRI image pair datasets. There are various metrics to quantify the fusion results, i.e., MS-SSIM (Wang et al., 2003), SCD (Aslantas and Bendes, 2015), Nabf (Kumar, 2013), HVS (Chen and Blum, 2009), and VIFF (Han et al., 2013). Multi-scale structural similarity (MS-SSIM) mainly measures the structural consistency between image blocks and has better expression ability compared to the general structural similarity. A higher MS-SSIM value indicates that the fused image is closer to the structural information of the source image, and the value range of MS-SSIM is [0, 1]. The sum of the correlations of differences (SCD) is an evaluation index based on the sum of correlations. A larger SCD value indicates that more source image information is contained in the fused image, and the value range of the SCD is [−2, +2]. Nabf measures how much noise and artificial information the algorithm introduces into the final fused image during fusion. Therefore, a smaller Nabf introduces less noise and the fusion quality of the image is better. The range of Nabf is [0, 1]. Visual information fidelity (VIFF) is a metric to evaluate the fused images based on visual information fidelity. A larger VIFF value indicates more fidelity of the fused image, and the value range of VIFF is [0, 1]. In addition, the best values of each metric in the given tables are highlighted in red.

#### 4.2.1 CT–MRI fusion

**FIGURE 4:**
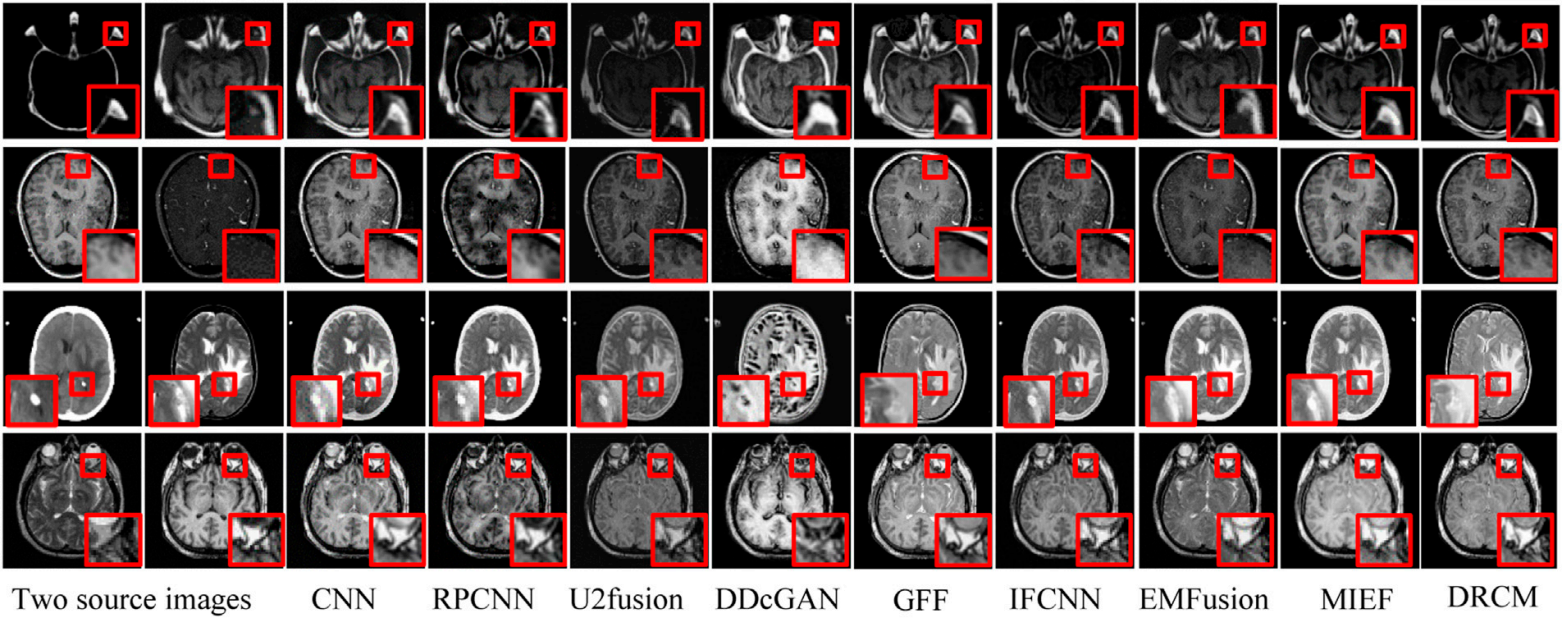
CT-MRI fusion images under different comparison methods. Two source images are reproduced with permission from http://www.med.harvard.edu/AANLIB/home.html.

The test set contains eight CT–MRI image pairs, and these images are widely used to test fusion models (Zhang et al., 2020; Huang et al., 2022b), of which four classic image pairs are used for qualitative comparison. Figure 4 shows the fusion images of the DRCM and eight comparison algorithms. For the four examples, our method almost has the clear boundaries of the tissues and organs, and the retention of details and the contrast of brightness are also good. In particular, RPCNN did not accurately extract complementary information between two source images, resulting in the introduction of noise into the fused image, as shown in the second example. DDcGAN cannot extract important features from the source images, which leads to less retention of multimodal information in the fused image, as shown in the first example. U2Fusion can slightly alleviate the problem of complementary information extraction but still encounters this problem to some extents, resulting in low contrast. The CNN has better performance in containing multimodal information, but it over-preserves the information from the first source image, resulting in blurred fusion results. The IFCNN alleviated the blurring problem but encounters low contrast. EMFusion has better performance in contrast but over-extracts the features of the second source image, which leads to the loss of important information of the first source image. MIEF has better performance in contrast and multimodal information retention but has blurred edges and contours in the fused image. In comparison, the DRCM has better performance showing clearer edges and contours, maintaining high contrast, and preserving more multimodal information from source images. It is worth noting that MIEF and the DRCM have richer details than other methods, which verifies the effectiveness of the disentangled representation network. Furthermore, compared with MIEF, the fusion images of the DRCM have clearer edges and contours, which suggests that our method has better fusion results. The fused images of the DRCM have clearer edges and contours and have significant advantages in expressing multimodal information, although the fused images slightly sacrifice the brightness of source images, which can be observed in the first and third examples. This is because the DRCM is used to solve the common redundancy problem in multimodal fusion processes and mainly focuses on the dynamic weighting of multimodal information by employing exclusive information, which may affect the expression of common visual information such as brightness.

**FIGURE 5:**
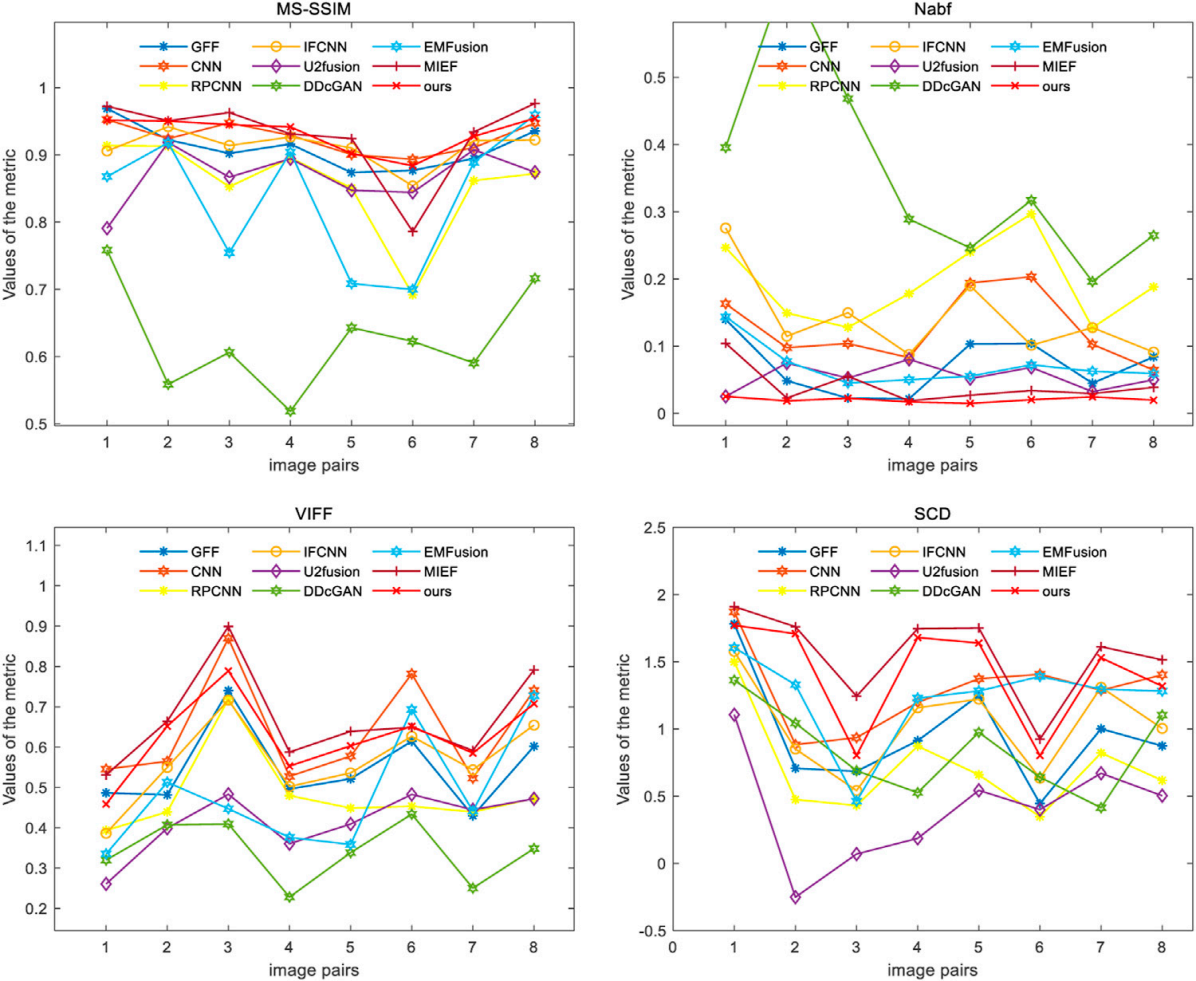
Results of four metrics of fusion images on CT–MRI image pairs under different methods.

Four evaluation metrics are used to quantitatively compare eight CT–MRI image pairs in the test set. Figure 5 and Table 1 represent line plots and average values of CT–MRI fusion results, respectively. The DRCM shows significant advantages in terms of fusion results, and the mean values of all the metrics are better than those obtained by the most comparison methods. The results are shown in Table 1. It is worth noting that MIEF is another work of ours, which uses the disentangled representation network for medical image fusion. The difference from this paper is that MIEF does not further process the subsequent disentangled features, while this paper uses two attentions to perform weighted fusion of those features. The experimental results show that MIEF and the DRCM based on the disentangled representation network obtain the best results, which proves the superiority of the disentangled representation method. In addition, the best values of the DRCM on Nabf and MS-SSIM also prove that the fusion images of the DRCM contain less noise and retain more structural information of the source images. Figure 5 shows that the DRCM achieves the best values of MS-SSIM and Nabf in almost all image pairs. In particular, the DRCM obtains the largest MS-SSIM values on image pairs 2 and 4 and the best values on almost all data pairs except for data pair 1. As for the SCD and VIFF, MIEF obtains the best average values, and the values of the DRCM fluctuated slightly. In general, these quantitative comparison results demonstrate the superiority of disentangled representation networks and the better performance of the DRCM. It is worth noting that the proposed DRCM is slightly worse than the traditional CNN and MIEF methods. Similar to Figure 4, the decrease in brightness may affect the visual quality of the fused image.

**TABLE 1:** Mean value and standard deviation of CT–MRI under different metrics.

Metrics	SCD	VIFF	MS-SSIM	Nabf
CNN	1.2948 ± 0.30	0.6412 ± 0.13	0.9257 ± 0.02	0.1266 ± 0.30
RPCNN	0.7158 ± 0.37	0.4806 ± 0.1	0.8564 ± 0.07	0.1944 ± 0.06
U2Fusion	0.4037 ± 0.41	0.4139 ± 0.07	0.868 ± 0.04	0.0544 ± 0.02
DDcGAN	0.8161 ± 0.34	0.3326 ± 0.07	0.6365 ± 0.08	0.3537 ± 0.01
GFF	0.9573 ± 0.41	0.5465 ± 0.1	0.9113 ± 0.03	0.0711 ± 0.04
IFCNN	1.0374 ± 0.35	0.5644 ± 0.1	0.9119 ± 0.02	0.1422 ± 0.06
EMFusion	1.2348 ± 0.33	0.4864 ± 0.15	0.8376 ± 0.1	0.0707 ± 0.03
MIEF	1.5579 ± 0.32	0.669 ± 0.12	0.9298 ± 0.06	0.0413 ± 0.03
DRCM	1.4067 ± 0.39	0.6249 ± 0.1	0.9321 ± 0.02	0.0204 ± 0.001

#### 4.2.2 PET–MRI fusion

**FIGURE 6:**
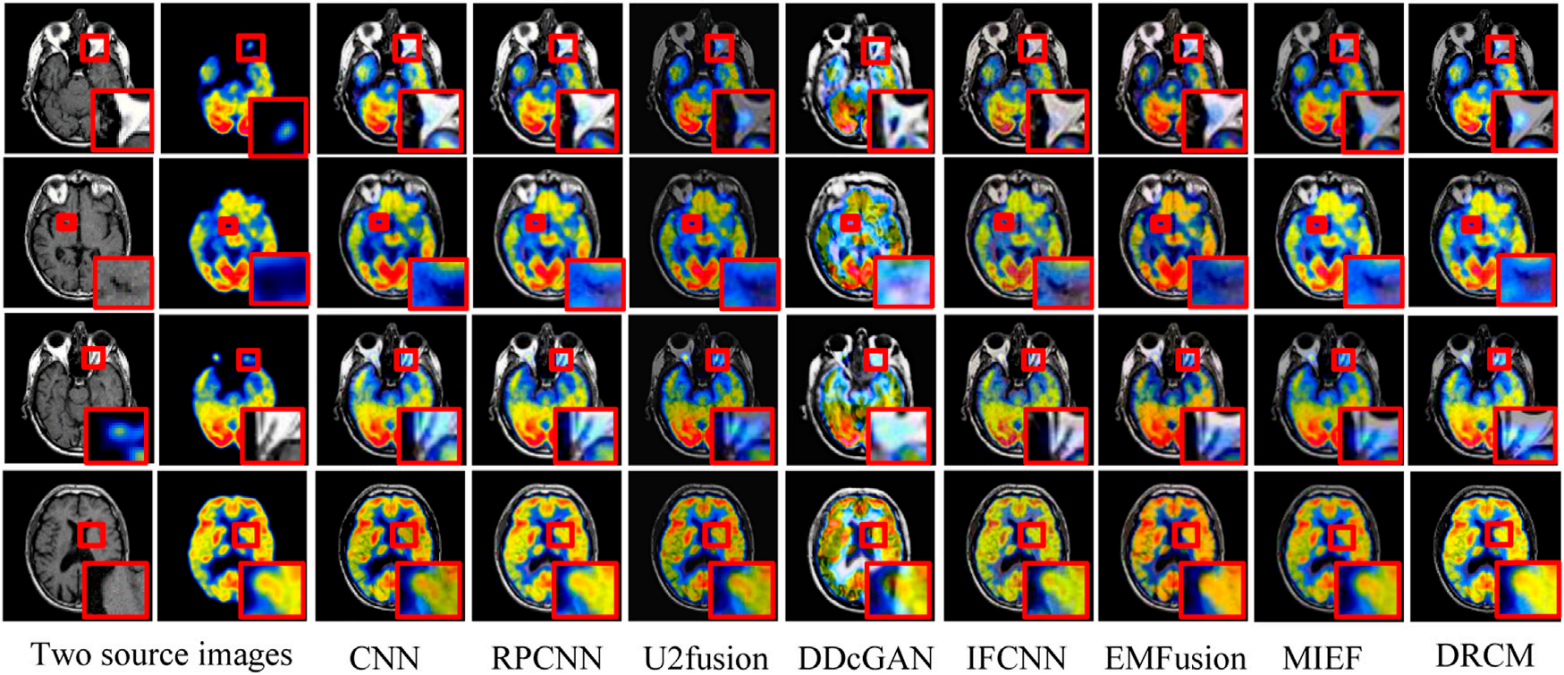
PET–MRI fusion images under different comparison methods. Two source images are reproduced with permission from http://www.med.harvard.edu/AANLIB/home.html.

Figure 6 represents the qualitative comparison results of different methods in PET–MRI image pairs. The results of the DRCM retain the color intensity of PET, while the contour details of MRI are very clear, and images have higher sharpness, fewer artifacts, and richer details, improving readability. Specifically, as shown in Figure 6, the CNN over-extracts anatomical information and loses functional information from PET images, resulting in color distortion in the fused image. RPCNN alleviates the issue of loss of functional information, but this issue also exists to a certain extent. U2Fusion better preserves the functional information but contains less anatomical information, resulting in a blurred fused image. DDcGAN cannot correctly extract multimodal complementary features, leading to undesirable artifacts in the fused image. The IFCNN over-preserves the anatomical information and lost functional information of the PET image. EMFusion slightly alleviates the problem of loss of functional features, but it also encounters this problem to some extent. MIEF better retains the functional information and anatomical information but has low contrast. Overall, our DRCM retains more functional information and clearer anatomical information than other comparative methods. However, it also has the disadvantage of insufficient brightness in PET–MRI fusion. As shown in the first example, although the fusion image of the DRCM retains more information, its anatomical information is not as bright as the source image. This phenomenon may be due to insufficient attention and processing of brightness information by the DRCM in the process of weighting multimodal information, and this direction will be an important object of our future research.

**FIGURE 7:**
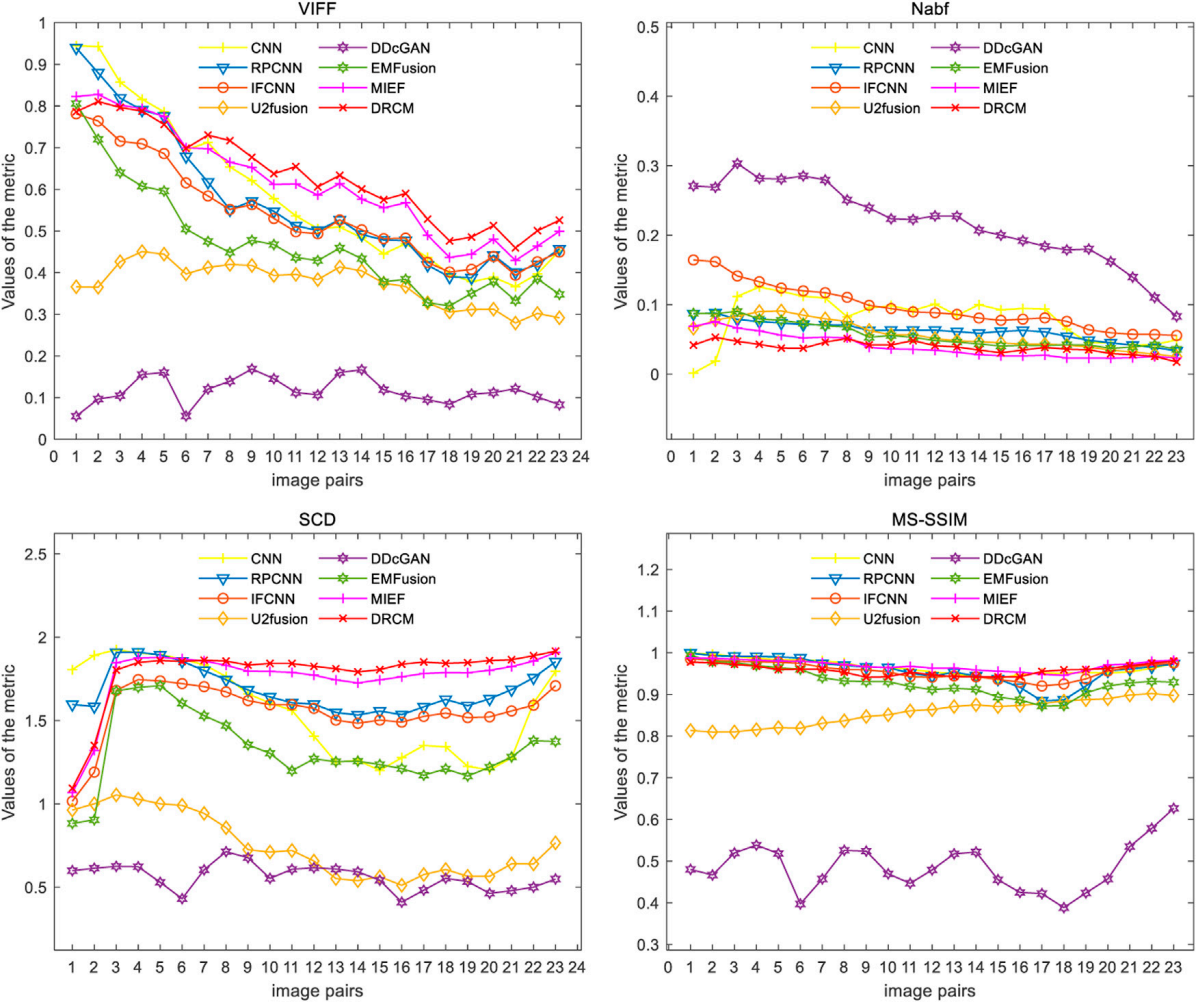
Results of the four metrics of fusion images on PET–MRI image pairs under different methods.

Figure 7 shows the comparison of the four metrics of the 23 typical image pairs with different methods, and the mean values of metrics under different methods are shown in Table 2. The experimental results show that our two works, namely, MIEF and the DRCM, obtain the best results in some metrics, which proves the superiority of the disentangled representation method. In addition, the best values of the DRCM on Nabf, SCD, and VIFF also prove that the fused image of the DRCM contains less noise compared to MIEF. The basic methods such as the CNN and RPCNN already have relatively good feature learning ability, but they also produce more noise during fusion, resulting in an unclear image outline. For a single image pair, our method also achieves good metric results on most of the image pairs. Specifically, for VIFF, the DRCM achieves the maximum average value and the maximum value on data pairs of 7–23. As for the SCD, the DRCM obtains the largest average value and largest value on image pairs of 6–23. Furthermore, the DRCM obtains the best average Nabf value and best values on data pairs of 3–8, while obtaining the suboptimal average value of MS-SSIM and largest values of image pairs of 17–19. MIEF obtains the best average MS-SSIM value; the better performance of MIEF and the DRCM demonstrates the advantages of the disentangled representation network. Through the comprehensive analysis of the actual image effects and indicators, the DRCM achieved better results compared to other methods.

**TABLE 2:** Mean value and standard deviation of PET–MRI under different metrics.

Metrics	SCD	Nabf	VIFF	MS-SSIM
CNN	1.5768 ± 0.28	0.0791 ± 0.03	0.5786 ± 0.18	0.9568 ± 0.02
RPCNN	1.6932 ± 0.13	0.0623 ± 0.01	0.5651 ± 0.16	0.9586 ± 0.03
U2Fusion	0.7513 ± 0.18	0.0566 ± 0.02	0.3658 ± 0.06	0.8583 ± 0.03
DDcGAN	0.5610 ± 0.07	0.2173 ± 0.06	0.1163 ± 0.03	0.4857 ± 0.05
IFCNN	1.5668 ± 0.17	0.0964 ± 0.03	0.5365 ± 0.11	0.9577 ± 0.02
EMFusion	1.3161 ± 0.21	0.0562 ± 0.02	0.4561 ± 0.13	0.9293 ± 0.03
MIEF	1.7657 ± 0.18	0.0388 ± 0.02	0.61 ± 0.0.12	0.9703 ± 0.01
DRCM	1.7974 ± 0.18	0.0371 ± 0.01	0.6282 ± 0.11	0.9595 ± 0.01

Table 3 shows the average time for different algorithms to process CT–MRI and PET–MRI datasets. It can be seen that CNN, RPCNN, U2Fusion, DDcGAN, and PMGI take several seconds or even more than 20 s, while IFCNN, EMFusion, MIEF, and DRCM only take less than 1 s. The processing time of the DRCM reaches 0.06 s, which ensures the fusion effect and the rapidity of imaging.

**TABLE 3:** Average time taken for different algorithms to process CT–MRI and PET–MRI datasets.

Methods	CNN (s)	RPCNN (s)	U2Fusion (s)	DDcGAN (s)	IFCNN (s)	EMFusion (s)	MIEF (s)	DRCM (s)
Time	21.341	12.337	8.5287	5.8927	0.0313	0.8881	0.0542	0.0624

#### 4.2.3 SPECT–MRI fusion

To further verify the generalization of the proposed method DRCM, we also conducted experiments to fuse SPECT and MRI image pairs.

**FIGURE 8:**
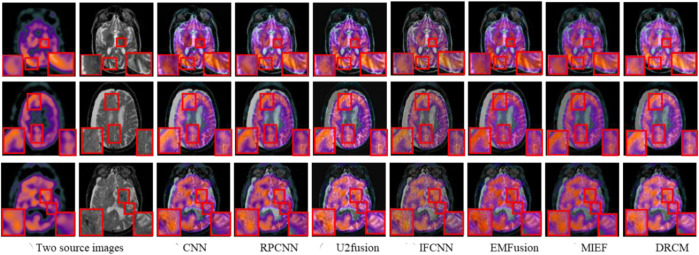
SPECT–MRI fusion images under different comparison methods. Two source images are reproduced with permission from http://www.med.harvard.edu/AANLIB/home.html.

Figure 8 represents the comparison results of the DRCM and other fusion algorithms on three typical image pairs. It can be intuitively seen that compared to other methods, the DRCM contains more functional information from SPECT, as well as more anatomical information. In addition, the DRCM also has a clearer edge. Specifically, the IFCNN cannot effectively extract functional features from SPECT, resulting in the loss of metabolic information and color distortion in fused images. EMFusion slightly alleviates color distortion issues but encounters it to some extent, which leads to a blurred fused image. RPCNN better retains functional information but has limited ability in extracting functional features, resulting in blurred contours of soft tissue. U2Fusion can effectively extract anatomical features from MRI while introducing white noise into the fused image. The CNN demonstrated superior soft tissue retention ability but lost some metabolic information, as shown in the first example. MIEF better contains the functional information but is not good at preserving anatomical information, resulting in blurred edges and contours of soft tissue. By comparison, the DRCM has better fusion performance, which not only contains more functional information but also has clearer edges and richer details. Therefore, these fusion results demonstrate that our method can also achieve better fusion results in the SPECT–MRI fusion. Similarly, the DRCM also limits the visual quality of the fused image due to little attention on visual information such as luminance.

**FIGURE 9:**
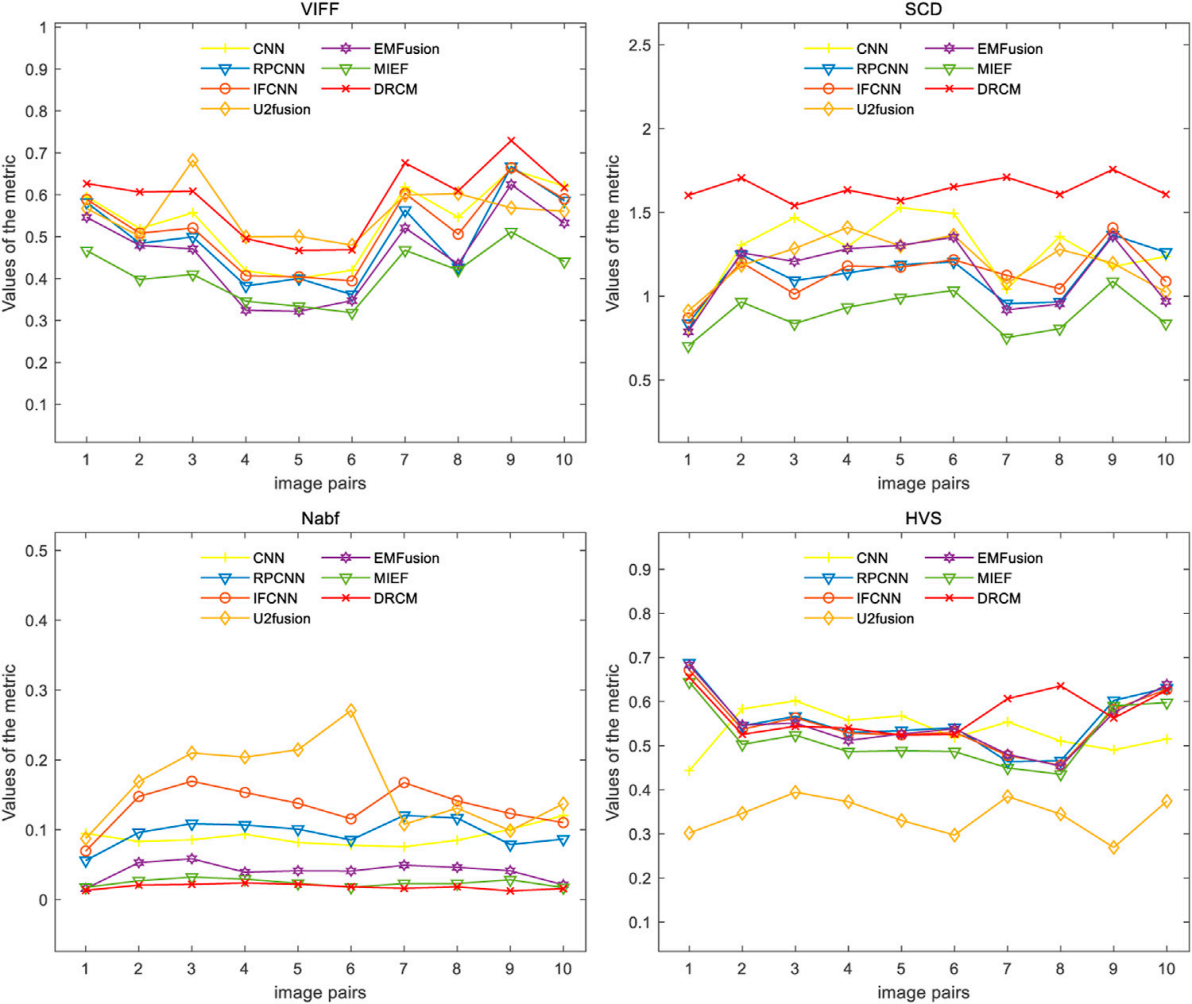
Results of the four metrics of fusion images on PET–MRI image pairs under different methods.

We also conducted quantitative experiments on this dataset, and the results of 10 typical image pairs are given in Figure 9 and Table 4. It can be seen that our method achieved the best results on all four indicators. Specifically, for SCD, the DRCM obtained the maximum average value and reached the maximum SCD value on all image pairs. For Nabf, the DRCM obtained the best average value and the best value on all data pairs except for 5 and 6. For VIFF, the DRCM obtained the largest average value and the maximum VIFF value on all data pairs except for 5 and 6. These results demonstrate that the DRCM not only has less noise and contains more source image information compared to other comparison methods but is also more consistent with the human visual system. Therefore, the DRCM has superior fusion performance compared to other comparison methods on the SPECT–MRI dataset.

**TABLE 4:** Mean value and standard deviation of SPECT–MRI under different metrics.

Metrics	SCD	Nabf	VIFF	HVS
CNN	1.2699 ± 0.22	0.0899 ± 0.01	0.5357 ± 0.09	0.5341 ± 0.04
RPCNN	1.126 ± 0.16	0.0957 ± 0.01	0.4952 ± 0.1	0.5566 ± 0.06
U2Fusion	1.203 ± 0.15	0.1630 ± 0.06	0.5561 ± 0.06	0.3416 ± 0.04
IFCNN	1.1319 ± 0.14	0.1336 ± 0.03	0.5187 ± 0.09	0.5498 ± 0.06
EMFusion	1.1394 ± 0.2	0.0407 ± 0.01	0.4602 ± 0.1	0.5504 ± 0.06
MIEF	0.895 ± 0.12	0.0239 ± 0.01	0.4113 ± 0.06	0.5204 ± 0.06
DRCM	1.6385 ± 0.06	0.0182 ± 0.01	0.5905 ± 0.08	0.5744 ± 0.05

Overall, for the fusion of SPECT–MRI, the DRCM has advantages in both qualitative and quantitative aspects compared with other methods. This result indicates that the proposed DRCM has satisfactory generalization ability.

## 5 Ablation analysis

Ablation experiments are carried out on three innovation points to test the effectiveness of the innovation points in the proposed method, including without the cross mutual information ablation experiment (w/o c-mi), the ablation experiment without coordinate attention (w/o coor), and the ablation experiment without multimodal attention (w/o multi). In the w/o c-mi ablation experiment, the network only extracts features and has no function to extract common and exclusive features. In the w/o coor ablation experiment, common and exclusive features of different modalities are not reinforced by coordinate attention. In the w/o multi ablation experiment, exclusive features are not dynamically weighted and highlighted by multimodal attention.

**FIGURE 10:**
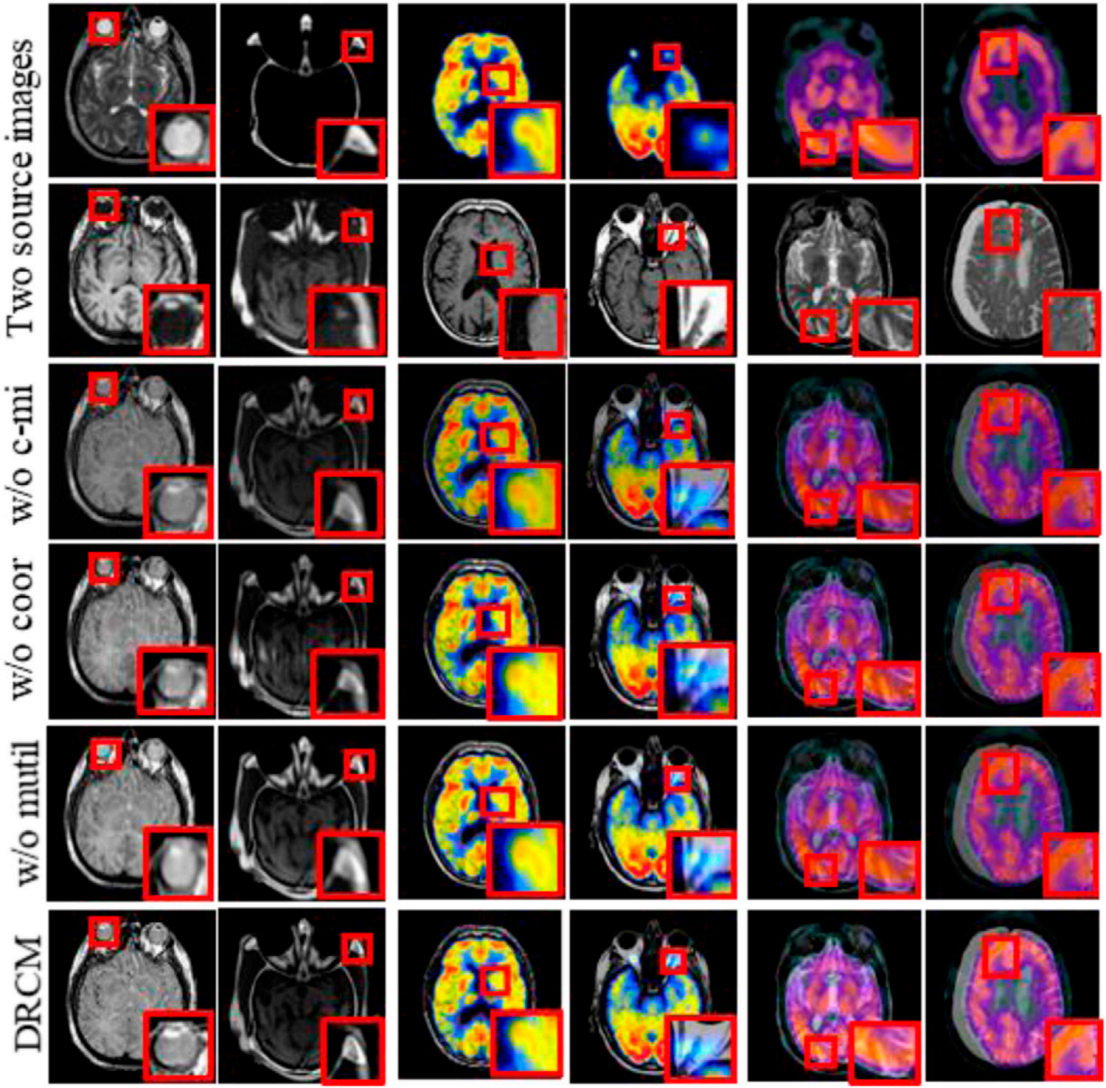
Fusion results of the ablation experiments. Two source images are reproduced with permission from http://www.med.harvard.edu/AANLIB/home.html.

CT–MRI, PET–MRI, and SPECT–MRI datasets are also used in ablation experiments, and Figure 10 shows the results of the experiments. Compared with the other control groups, the fusion results of the DRCM are the best, and the image texture is clearer, the contrast is higher, and the details are richer. The fused images of w/o c-mi and w/o coor have many artifacts, and the results of w/o multi are greatly improved compared with those of w/o c-mi and w/o coor. This shows that cross mutual information and coordinate attention play an important role in removing artifacts and improving image quality. Compared with w/o multi, our method obtains fusion images with clearer contours, indicating that multimodal attention is helpful for the expression of exclusive features of modalities.

Tables 5–7 show the quantitative comparison results of ablation experiments, respectively, from left to right by row; the indicator data gradually become better, and our method achieves the optimal results. Among them, the w/o multi greatly improved, which indicates that the cross mutual information and coordinate attention greatly increase the quality of the fused image by extracting and enhancing common and exclusive features. However, in the w/o coor and w/o multi ablation experiments, the values of Nabf increase significantly, which indicates that coordinate attention and multimodal attention can suppress the generation of noise and eliminate artifacts during fusion. In summary, the results of ablation experiments show the necessity and importance of the three innovations included in our method.

**TABLE 5:** Mean value and standard deviation of four metrics for ablation experiments on the CT–MRI dataset.

Metrics	w/o c-mi	w/o coor	w/o multi	DRCM
SCD	0.83 ± 0.33	1.1472 ± 0.38	1.3247 ± 0.43	1.4067 ± 0.39
VIFF	0.4838 ± 0.08	0.505 ± 0.08	0.6 ± 0.1	0.6249 ± 0.1
MS-SSIM	0.8765 ± 0.03	0.8288 ± 0.08	0.9016 ± 0.05	0.9321 ± 0.02
Nabf	0.0235 ± 0.02	0.072 ± 0.04	0.041 ± 0.02	0.0204 ± 0.001

**TABLE 6:** Mean value and standard deviation of four metrics for ablation experiments on the PET–MRI dataset.

Metrics	w/o c-mi	w/o coor	w/o multi	DRCM
SCD	1.13 ± 0.17	1.3667 ± 0.14	1.6178 ± 0.19	1.7974 ± 0.18
VIFF	0.4161 ± 0.07	0.4479 ± 0.08	0.5403 ± 0.08	0.6282 ± 0.11
MS-SSIM	0.8884 ± 0.02	0.9045 ± 0.01	0.9435 ± 0.01	0.9595 ± 0.01
Nabf	0.015 ± 0.01	0.057 ± 0.03	0.045 ± 0.02	0.0371 ± 0.01

**TABLE 7:** Mean value and standard deviation of four metrics for ablation experiments on the SPECT–MRI dataset.

Metrics	w/o c-mi	w/o coor	w/o multi	DRCM
SCD	0.3109 ± 0.14	0.8675 ± 0.1	0.8145 ± 0.09	1.6385 ± 0.06
VIFF	0.2675 ± 0.05	0.4005 ± 0.05	0.3914 ± 0.06	0.5905 ± 0.08
MS-SSIM	0.8137 ± 0.02	0.8893 ± 0.01	0.8818 ± 0.01	0.9363 ± 0.01
Nabf	0.0245 ± 0.01	0.0259 ± 0.01	0.034 ± 0.01	0.0182 ± 0.01

This paper mainly explores the common redundancy issues and designs dynamic weights for multiple modalities to accurately reflect the importance of different modalities. However, the method that concentrates on multimodal dynamic weighting has limitations on the expression of visual information such as brightness. Therefore, in future, we will achieve multimodal preservation of more source image information while further improving the visual quality of fused images.

## 6 Conclusion and recommendations

In this article, a disentangled representation medical image fusion network with coordinated attention and multimodal attention is proposed. In this network, the common features and exclusive features of each modality pair are obtained by cross mutual information and adversarial objective, respectively. Then, the common features and exclusive features of different modes are enhanced by coordinating attention, and the exclusive features are weighted by multimodal attention. Finally, the two processing features are fused by the elementwise-maximum method. The ablation experiment results show that the innovation points in the method significantly increase the effectiveness of the fused network. In addition, the network is tested with CT–MRI, PET–MRI, and SPECT–MRI pairs in the experiments. Eight state-of-the-art comparison methods and four metrics are adopted for qualitative and quantitative analyses, and the comprehensive results show that the fused images obtained by the DRCM have better performance.

It is worth noting that some challenges arose in the research process, such as slight insufficiency in the brightness of fused images. Therefore, addressing this limitation is the future direction and recommendation. In addition, to the best of our knowledge, this is the first time a multimodal attention mechanism was applied to medical image fusion and achieved better results, which can be further extended to other image fusion tasks. The medical images used in this paper are all registered, which is not common in daily life. Next, we will consider registration and fusion of common images, and images of other specific diseases will also be considered for fusion studies.

## Data Availability

The original contributions presented in the study are included in the article/Supplementary Material; further inquiries can be directed to the corresponding author.
